# Structure of the Discoidin Domain Receptor 1 Extracellular Region Bound to an Inhibitory Fab Fragment Reveals Features Important for Signaling

**DOI:** 10.1016/j.str.2012.02.011

**Published:** 2012-04-04

**Authors:** Federico Carafoli, Marie Cathrin Mayer, Kazushige Shiraishi, Mira Anguelova Pecheva, Lai Yi Chan, Ruodan Nan, Birgit Leitinger, Erhard Hohenester

**Affiliations:** 1 Department of Life Sciences, Imperial College London, London SW7 2AZ, UK; 2 National Heart and Lung Institute, Imperial College London, London SW7 2AZ, UK; 3 Institute of Structural and Molecular Biology, University College London, London WC1E 6BT, UK

## Abstract

The discoidin domain receptors, DDR1 and DDR2, are constitutively dimeric receptor tyrosine kinases that are activated by triple-helical collagen. Aberrant DDR signaling contributes to several human pathologies, including many cancers. We have generated monoclonal antibodies (mAbs) that inhibit DDR1 signaling without interfering with collagen binding. The crystal structure of the monomeric DDR1 extracellular region bound to the Fab fragment of mAb 3E3 reveals that the collagen-binding discoidin (DS) domain is tightly associated with the following DS-like domain, which contains the epitopes of all mAbs. A conserved surface patch in the DS domain outside the collagen-binding site is shown to be required for signaling. Thus, the active conformation of the DDR1 dimer involves collagen-induced contacts between the DS domains, in addition to the previously identified association of transmembrane helices. The mAbs likely inhibit signaling by sterically blocking the extracellular association of DDR1 subunits.

## Graphical abstract

**Graphical Abstract:**

**Figure:**
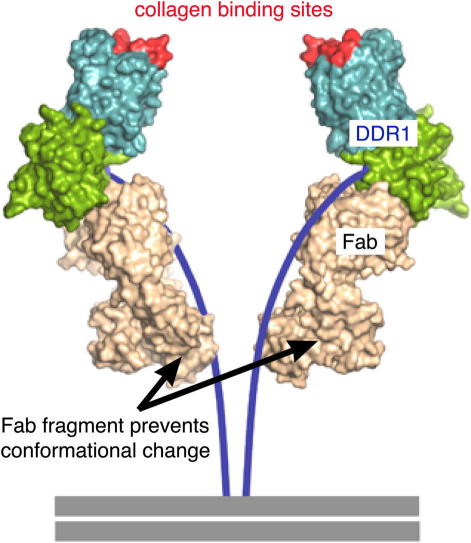

**Highlights:**

► Monoclonal antibodies inhibit DDR1 signaling without blocking collagen binding ► The DDR1 extracellular region consists of a DS and a DS-like domain ► The collagen-binding DS domain contains a patch that is essential for signaling ► The mAbs bind to the DS-like domain, preventing formation of the active DDR dimer

## Introduction

Receptor tyrosine kinases (RTKs) control many fundamental cellular processes, such as cell proliferation, differentiation, migration, and metabolism (Lemmon and Schlessinger, 2010). RTK activity is normally tightly controlled, and dysregulation of RTK activity is associated with many human cancers and other pathologies. Ligand binding to the extracellular region of RTKs leads to autophosphorylation of their cytoplasmic kinase domains, creating docking sites for effectors of downstream signaling. The two major strategies for controlling unwanted RTK activity in human patients are inhibition by monoclonal antibodies (mAbs) directed against their extracellular regions or by small molecules targeting the kinase active site (Adams and Weiner, 2005; Gschwind et al., 2004).

The discoidin domain receptors, DDR1 and DDR2, are RTKs that are activated by several types of triple-helical collagen, a major component of the animal extracellular matrix (Leitinger, 2011; Shrivastava et al., 1997; Vogel et al., 1997). The DDRs are widely expressed in mammalian tissues and have important roles in embryo development and human disease (Vogel et al., 2006). For example, DDR1 is essential for mammary gland development (Vogel et al., 2001), and DDR2 is essential for the growth of long bones (Labrador et al., 2001). DDR2 mutations in humans cause a rare, severe form of dwarfism (Ali et al., 2010; Bargal et al., 2009). The DDRs are also implicated in cancer, fibrotic diseases, atherosclerosis, and arthritis (Vogel et al., 2006). Mechanistically, the DDRs have several features that distinguish them from other RTKs. Compared with the rapid response of typical RTKs to their soluble ligands (e.g., growth factors), collagen-induced DDR autophosphorylation is slow and sustained (Shrivastava et al., 1997; Vogel et al., 1997). Furthermore, Src kinase plays an essential role in DDR activation (Ikeda et al., 2002).

Both DDRs are composed of an N-terminal discoidin (DS) domain (Baumgartner et al., 1998), followed by a predicted DS-like domain (our unpublished results; Lemmon and Schlessinger, 2010), an extracellular juxtamembrane (JM) region, a transmembrane (TM) helix, a large cytosolic JM region, and a C-terminal tyrosine kinase domain. Collagen binds to the DS domain, and the structural determinants of the DDR-collagen interaction have been extensively studied (Carafoli et al., 2009; Ichikawa et al., 2007; Konitsiotis et al., 2008; Leitinger, 2003; Xu et al., 2011). The remainder of the extracellular region has not been characterized structurally or functionally.

How collagen binding results in DDR activation is a major unresolved question. DDR1 can be activated by short collagen-like peptides, showing that DDR clustering by multivalent collagen assemblies (e.g., fibrils) is not essential for activation (Konitsiotis et al., 2008). The DDRs are constitutive dimers at the cell surface, and residues within the TM helix are required for signaling (Noordeen et al., 2006). In fact, a comprehensive analysis has shown that the DDRs have the highest propensity of TM helix self-interactions in the entire RTK superfamily (Finger et al., 2009). Therefore, the conformational changes resulting from collagen binding are likely to occur in the context of a stable DDR dimer. Our crystal structure of a DDR2 DS-collagen peptide complex (Carafoli et al., 2009) revealed a 1:1 complex and did not clarify how collagen binding affects the conformation of the DDR dimer. Here, we report the functional characterization of a set of inhibitory anti-DDR1 mAbs and the crystallization of the almost complete extracellular region of DDR1 bound to a mAb Fab fragment. The crystal structure led to the discovery of DDR1 residues that are required for signaling, even though they are not part of the known collagen-binding site. These results provide insight into the process of DDR1 activation.

## Results

### Generation and Characterization of Anti-DDR1 mAbs

**Figure 1:**
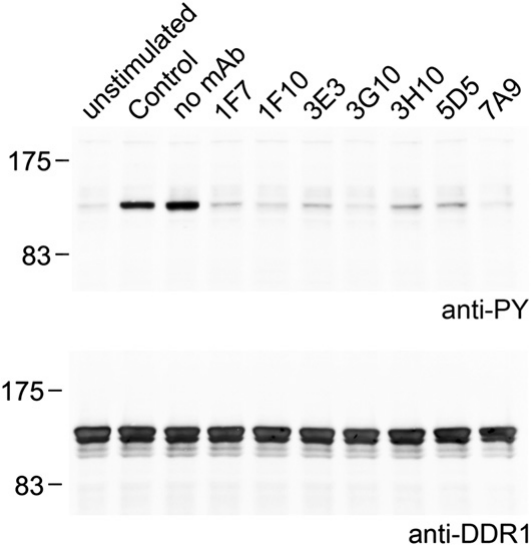
The Anti-DDR1 mAbs Block Collagen-Induced DDR1 Phosphorylation DDR1b was transiently expressed in HEK293 cells, and the cells were stimulated with 10 μg/ml collagen I in the absence or presence of 10 μg/ml of the indicated anti-DDR1 mAbs. Aliquots of cell lysates were analyzed by SDS-PAGE and western blotting. The blots were probed with anti-phosphotyrosine (anti-PY) mAb 4G10 (upper blot) and reprobed with anti-DDR1 Ab (lower blot). Control, mouse IgG1 isotype control Ab. The experiment was performed three times with similar results. See also Figure S1.

**Figure 2:**
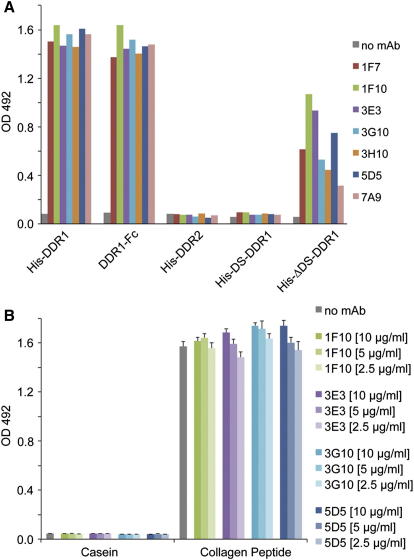
The Anti-DDR1 mAbs Bind to the DS-Like Domain and Do Not Inhibit Ligand Binding (A) ELISA showing binding of the indicated anti-DDR1 mAbs to recombinant DDR proteins immobilized on 96-well plates. Shown is a representative of three independent experiments, each performed in duplicate. (B) Solid-phase binding assay with recombinant DDR1-Fc protein added to 96-well plates coated with either casein or collagen peptide III-23 (Xu et al., 2011). DDR1-Fc was preincubated with the indicated anti-DDR1 mAbs before addition to the wells. Bound DDR1-Fc was detected with anti-human Fc Ab and was measured as absorbance at 492 nm. Shown is a representative of three independent experiments, each performed in triplicate. The error bars indicate the sample standard deviation (n = 3).

We immunized mice with a recombinant protein spanning the entire extracellular region of human DDR1 and obtained seven anti-DDR1 mAbs. All seven mAbs were found to inhibit the collagen-induced autophosphorylation of DDR1 expressed in HEK293 cells (Figure 1), and this inhibitory activity was retained by Fab fragments generated from five of the seven mAbs (Figure S1 available online). Dose-dependent inhibition experiments revealed no substantial differences in activity among the mAbs, which all reduced DDR1 phosphorylation to background levels when applied at 2 μg/ml, but were only partially inhibitory at 0.2 μg/ml (data not shown). Similarly, an enzyme-linked immunosorbent assay (ELISA) with the immobilized DDR1 extracellular region (His-DDR1) showed that all mAbs bound with comparable apparent dissociation constants of ∼2 nM (data not shown). In an ELISA with domain deletion constructs, all seven mAbs bound to the membrane-proximal DS-like domain (His-ΔDS-DDR1), but not to the membrane-distal DS domain containing the collagen-binding site (His-DS-DDR1) (Figure 2A). This result suggested that the inhibitory activity of the mAbs on cells was unlikely to be the result of a block of collagen binding. Indeed, when we tested a subset of mAbs in a direct collagen binding assay, we found that binding of DDR1-Fc to a high-affinity collagen peptide (III-23) (Xu et al., 2011) was not affected by the addition of mAbs (Figure 2B). The combined results demonstrate that the anti-DDR1 mAbs inhibit DDR1 function by interfering with the signal transduction process resulting from collagen binding.

### Crystal Structure of a DDR1-Fab Complex

For several years, we had attempted unsuccessfully to obtain diffracting crystals of the extracellular regions of DDR1 or DDR2. Fab fragments of mAbs have been instrumental in facilitating the crystallization of many recalcitrant proteins (Nettleship et al., 2008). We therefore screened the Fab fragments of six anti-DDR1 mAbs for complex formation and cocrystallization with the extracellular region of DDR1 (the 1F7 Fab could not be used because it aggregated in solution). Because the His-DDR1 construct used for mAb generation included the JM region that is predicted to be unstructured, we produced a construct terminating at the predicted C terminus of the DS-like domain, Asp367. All Fab fragments bound to this shortened DDR1 construct, as determined by analytical size exclusion chromatography (data not shown). We obtained crystals of the DDR1-3E3 Fab complex and determined its structure at a resolution of 2.8 Å (Table 1).

**Figure 3:**
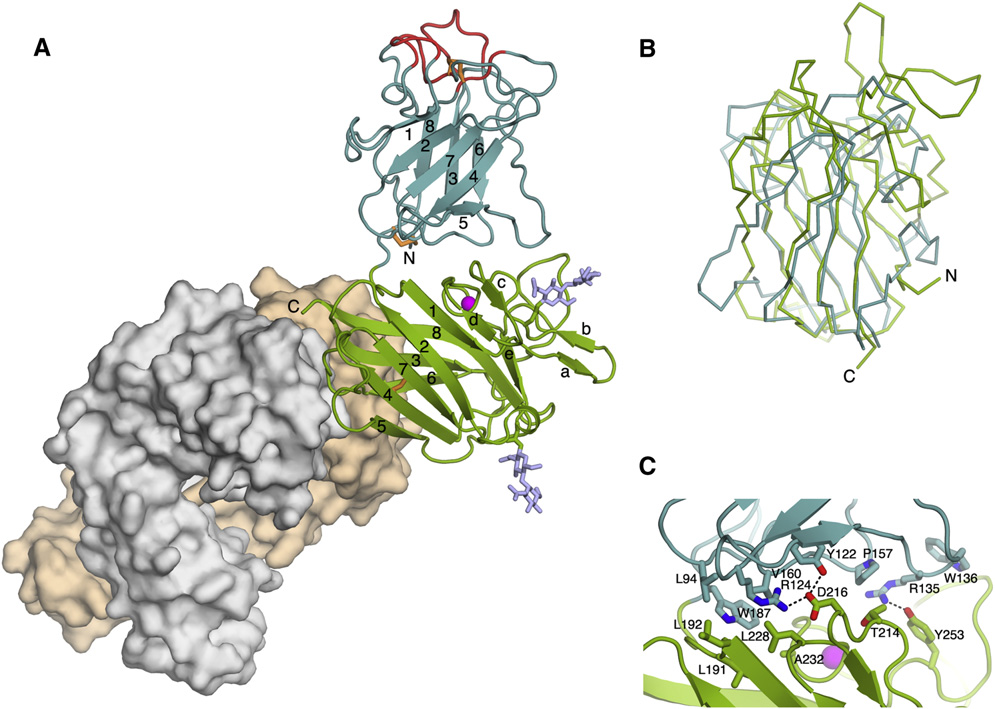
Crystal Structure of the DDR1-3E3 Fab Complex (A) Overall structure. The 3E3 Fab fragment is shown as a surface (tan, light chain; gray, heavy chain), and DDR1 is shown as a cartoon (cyan, DS domain; green, DS-like domain; and red, collagen-binding loops; Carafoli et al., 2009; orange, disulphide bridges). A calcium ion is shown as a magenta sphere and the two *N*-linked glycans are shown as light blue sticks. The N and C termini of the DDR1 construct are indicated. The β strands of the jelly roll in the DS and DS-like domains are numbered 1–8, and the extra β strands in the DS-like domain are labeled a–e. (B) Superposition of the DS domain (cyan) and the DS-like domain (green) of DDR1. (C) Detailed structure of the interface between the DS domain (cyan) and the DS-like domain (green) in DDR1. Selected residues are shown in atomic detail and labeled. Hydrogen bonds are indicated by dashed lines. See also Figures S2 and S3.

The extracellular region of DDR1 revealed by the crystal structure is a compact structure measuring approximately 70 × 50 × 40 Å (Figure 3A). The DS and DS-like domains are arranged such that the long axes of their β-barrels are roughly perpendicular to each other. There is an extensive interface between the two domains that buries 1410 Å^2^ of solvent-accessible surface (i.e., the interface measures ∼700 Å^2^). The domain arrangement in DDR1 is reminiscent of the tandem DS domains in neuropilins (Appleton et al., 2007; Vander Kooi et al., 2007), but the second domain is rotated differently in the two proteins (Figure S2). The N and C termini of the crystallized DDR1 construct are located on the same face of the molecule near the interdomain linker. In the intact receptor, the C terminus of the crystallized construct would be linked to the TM helix by the 50-residue JM region. The DDR1 DS domain is very similar to the DDR2 DS domain (rmsd of 0.61 Å for 156 Cα atoms), which was previously crystallized in complex with a collagen-like peptide (Carafoli et al., 2009). The collagen-binding loops of the DDR1 DS domain, which are opposite the DS-like domain, have weak electron density and high temperature factors, suggesting that they are quite mobile in the absence of the collagen ligand. The 3E3 Fab fragment is bound near the C terminus of the DS-like domain, distant from the collagen-binding site (for a description of the epitope, see below).

### Structure of the DS-like Domain

As predicted (our unpublished results; Lemmon and Schlessinger, 2010), the DS-like domain belongs to the coagulation factor V/VIII type C superfamily. A search with the program SSM (Krissinel and Henrick, 2004) showed that the DS-like domain of DDR1 is most closely related to family 32 carbohydrate-binding modules (CBMs) (Boraston et al., 2004), but a pairwise alignment of the DS and DS-like domains of DDR1 gave only a marginally lower Z-score and a rmsd of 3.0 Å for 120 aligned Cα atoms (Figure 3B). To facilitate the comparison of the DS and DS-like domains, the eight β strands that are common to both domains have been labeled β1–β8. The DS-like domain contains five additional strands, labeled βa–βe, in a long insertion between β1 and β2. Both domains are characterized by two antiparallel β sheets with jellyroll topology (β1-β2-β7-β4 sheet and β5-β6-β3-β8 sheet). At one end of the β-barrel (the “bottom”) the β2–β3 and β6–β7 loops cross over between the sheets and create a relatively flat surface. At the other end (the “top”), several long and irregular loops protrude from the barrel. In the DS domain, these loops constitute the collagen-binding site (Carafoli et al., 2009; Ichikawa et al., 2007; Leitinger, 2003). In the DS-like domain, they contribute the extra strands βa–βe, two *N*-linked glycosylation sites (Asn211 and Asn260), and a calcium-binding site. The calcium ion is coordinated by the side chains of Asp233 and Glu361, as well as by three main chain carbonyl groups; an analogous calcium coordination is seen in many family 32 CBMs (Boraston et al., 2004). It is noteworthy that the glycosylation site at Asn211 and the calcium ligands are strictly conserved in all vertebrate DDRs (Figure S3). A second ion in the DS-like domain was also modeled as calcium, but this ion appears to be bound more weakly and may be a crystal artifact (not shown). The DS-like domain of DDR1 contains three cysteines: Cys303 and Cys348, which form a deeply buried disulphide bridge linking the adjacent β4 and β7 strands, and Cys287, which is unpaired and also buried. A previous study suggested that Cys303 and Cys348 may be involved in the covalent dimerization of DDR1 (Abdulhussein et al., 2008). However, the DS-like domain would have to unfold for these two residues to become available for intermolecular disulphide bridges. The disulphide-linked dimers seen in cell lysates in that study therefore are more likely to have resulted from oxidation following denaturation.

The interface between the DS and DS-like domains of DDR1 is formed between the bottom of the DS domain, in particular the β4–β5 and β6–β7 loops, and the long convoluted insertion between strands β1 and β2 of the DS-like domain (Figure 3C). A key interface residue is Trp187, which is located in the short linker between the two domains and which interacts with residues of both the DS domain (Leu94 and Val160) and the DS-like domain (Leu191, Leu192, Leu228, and Ala232). With the exception of Leu192 and Ala232, these residues are strictly conserved in all vertebrate DDRs (Figure S3). Also conserved is an ion pair spanning the interface, involving Arg124 of the DS domain and Asp216 of the DS-like domain. Additional interdomain contacts are made between the 134–138 and 245–253 loops (Figure 3C). Even though the shape complementarity of the domain interface is not particularly high (sc-value of 0.56; Lawrence and Colman, 1993), its size and the conservation of key interface residues suggest that the domain arrangement seen in our structure is stable and representative of DDRs in general.

### DS Domain Residues Required for DDR1 Signaling

**Figure 4:**
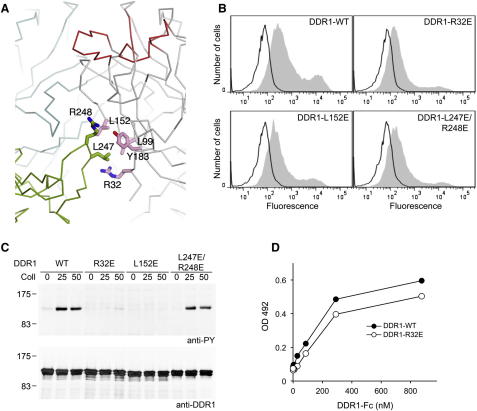
A Conserved Patch in the DS Domain Is Required for DDR1 Signaling (A) The lattice contact resulting in a symmetric DDR1 dimer (see text). The DDR1 molecule on the left is in cyan (DS domain) and green (DS-like domain); the DDR1 molecule on the right is in gray, with the collagen-binding loops (Carafoli et al., 2009) in red. The 2-fold symmetry axis is vertical. Selected residues are shown in atomic detail (pink, conserved surface patch in the DS domain). (B) Cell surface expression of mutants. Wild-type DDR1b or the indicated mutants were transiently expressed in HEK293 cells. The cells were stained on ice with 10 μg/ml of anti-DDR1 mAb 7A9 (filled gray histograms) or mouse IgG1 isotype control Ab (black lines) followed by FITC-conjugated goat-anti mouse IgG and analysis by flow cytometry. The experiment was performed twice with similar results. (C) Collagen-induced activation of mutants. Wild-type DDR1b or the indicated mutants were transiently expressed in HEK293 cells. The cells were stimulated with collagen I at the indicated concentrations (in μg/ml). Aliquots of cell lysates were analyzed by SDS-PAGE and western blotting. The blots were probed with anti-phosphotyrosine (anti-PY) mAb 4G10 (upper blot) and reprobed with anti-DDR1 Abs (lower blot). The experiment was performed three times with similar results. (D) Solid-phase binding assay with recombinant DDR1-Fc protein (filled circles, wild-type; open circles, R32E mutant) added to 96-well plates coated with collagen peptide III-23 (Xu et al., 2011). Bound DDR1-Fc was detected with anti-human Fc Ab and was measured as absorbance at 492 nm. Shown is a representative of two independent experiments, each performed in duplicate.

The DDRs are believed to be constitutive dimers at the cell surface (Mihai et al., 2009; Noordeen et al., 2006). Analytical ultracentrifugation of the crystallized DDR1 ectodomain construct showed it to be monomeric at concentrations of up to 6.8 mg/ml (data not shown), in agreement with an earlier result obtained by size-exclusion chromatography for His-DDR1 (Leitinger, 2003). However, because the high protein concentration in the crystal might favor a very weak dimer association, we inspected the crystal lattice for DDR1 dimers. There was only one plausible dimer. The interface between the two DDR1 molecules in this dimeric lattice contact is dominated by two identical, symmetry-related contacts and buries 1580 Å^2^ of solvent-accessible surface with good shape complementarity (sc-value of 0.64). In each of the symmetry-related contacts, four DS domain residues—Arg32, Leu99, Leu152, and Tyr183—interact with Leu247 and Arg248 of the other DDR1 molecule (Figure 4A). The four DS domain residues in the contact are strictly conserved in DDR1 and DDR2 from several species, whereas Leu247 and Arg248 are variable (Figure S3). To test whether these DDR1 regions are required for function, we expressed three DDR1 mutants (R32E, L152E, and L247E/R248E) in HEK293 cells and measured their collagen-induced autophosphorylation. Flow cytometry showed that the mutants were expressed at the cell surface similarly to wild-type DDR1 (Figure 4B). Furthermore, SDS-PAGE analysis of the mutants showed the characteristic two bands corresponding to the immature (intracellular) and mature (cell surface-expressed) glycoforms of the receptor (Noordeen et al., 2006) (Figure 4C). The L247E/R248E double mutation had no effect on DDR1 activation (Figure 4C), indicating that the dimeric crystal lattice contact does not recapitulate a signaling state of the receptor. In sharp contrast, the R32E and L152E mutations abrogated DDR1 activation (Figure 4C). This result was unlikely to be due to an effect on ligand binding, given that the two mutations are distant from the high-affinity collagen-binding site (Carafoli et al., 2009). Indeed, collagen-binding experiments with soluble DDR1 R32E and DDR2 L152E proteins (Figure 4D and data not shown) confirmed that these mutants are not defective in collagen binding. These findings demonstrate that the conserved surface patch in the DS domain containing Arg32 and Leu152 is required for DDR1 signaling, even though it is not part of the primary collagen-binding site.

### Epitopes of Anti-DDR1 mAbs

**Figure 5:**
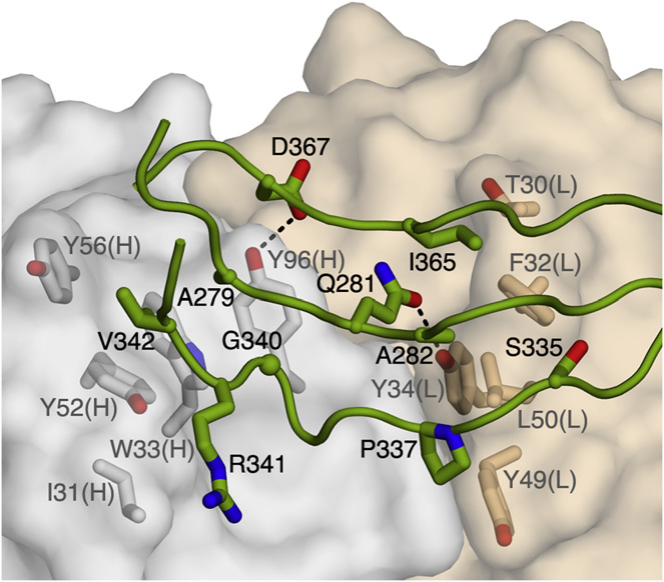
Detailed Structure of the DDR1-3E3 Fab Interface The 3E3 Fab fragment is shown as a semitransparent surface (tan, light chain; gray, heavy chain), and the DDR1 region interacting with the Fab is shown as a green cartoon. Selected interface residues are shown in atomic detail and labeled. Hydrogen bonds are indicated by dashed lines.

The 3E3 epitope is formed from three regions of the DDR1 DS-like domain, which are discontinuous in sequence but contiguous in space: the start of β3 (Ala279, Gln281, and Ala282), the β6–β7 loop (Ser 335, Pro337, Gly340, Arg341, and Val342), and the very end of the DS-like domain (Ile365 and Asp367) (Figure 5). The 3E3 Fab uses predominantly aromatic residues to recognize this epitope: Thr30, Phe32, Tyr34, Tyr49, and Leu50 of the light chain; and Ile31, Trp33, Tyr52, Tyr56, and Tyr96 of the heavy chain. In total, the DDR1-3E3 interface buries 1390 Å^2^ of solvent-accessible surface and has a high shape complementarity value of 0.68, a typical value for antibody-antigen complexes (Lawrence and Colman, 1993). The finding that mAb 3E3 binds close to the C terminus of the DS-like domain suggests that it may inhibit DDR1 function by preventing the association of the DS-like domains and/or JM regions in the signaling DDR1 dimer. The JM region is unlikely to make a major contribution to 3E3 binding, however, given that the 3E3 Fab and the truncated DDR1 ectodomain construct used for crystallization form a stable complex upon size-exclusion chromatography (see Experimental Procedures).

**Figure 6:**
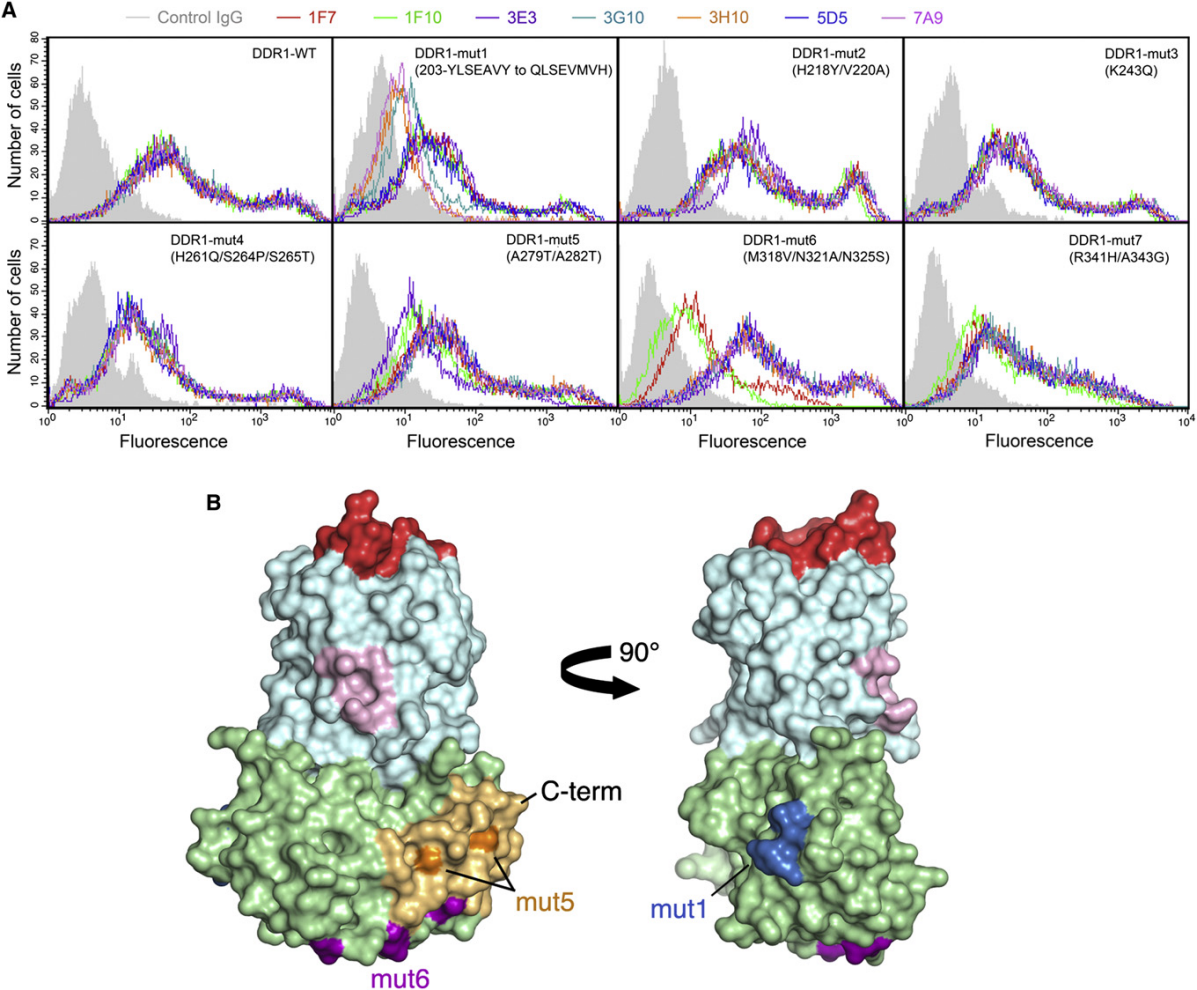
Epitope Mapping of Anti-DDR1 mAbs (A) Wild-type DDR1b or the indicated mutants were transiently expressed in HEK293 cells. The cells were stained on ice with 10 μg/ml of the indicated anti-DDR1 mAbs or mouse IgG1 isotype control Ab, followed by FITC-conjugated goat-anti mouse IgG and analysis by flow cytometry. Binding of isotype control Ab is shown by the filled gray histograms. Shown are representative data of at least three experiments for each DDR1 mutant. (B) Surface representation of DDR1 structure showing the location of mAb epitopes determined by mutation (mut1, blue: 3G10, 3H10, 7A9; mut6, purple: 1F7, 1F10). The 3E3 footprint from the crystal structure is shown in light orange, and mut5 is shown in dark orange. The DS and DS-like domains are in cyan and green, respectively. The collagen-binding site and conserved surface patch (Arg32, Leu99, Leu152, and Tyr183) are shown in red and light pink, respectively. The C terminus is indicated. See also Figure S4.

To better understand how the anti-DDR1 mAbs inhibit DDR1 function, it was of interest to determine the epitopes of the other mAbs as well. We therefore made a series of DDR1 mutants, which targeted all linear and nonconservative human-to-mouse substitutions in the DS-like domain (Figure S3). These DDR1 mutants were expressed in HEK293 cells, and mAb binding was measured by flow cytometry (Figure 6A and Figure S4). Mutant mut7 (R341H/A343G) was consistently expressed at lower levels than the wild-type protein, suggesting that the mutation may have compromised the DDR1 structure. All other mutants were expressed at similar levels to the wild-type protein. Saturating concentrations of all but one mAbs gave similar fluorescence profiles for wild-type DDR1. The single exception was 1F10, which is a different isotype (IgG2b) than the other anti-DDR1 mAbs (IgG1) and therefore may be detected less well by the secondary Ab.

Four of the DDR1 mutants (mut2, mut3, mut4, and mut7) showed unperturbed binding of all seven mAbs. The mut1 mutation (203-YLSEAVY to QLSEVMVH) abolished binding of mAbs 3G10, 3H10, and 7A9. The mut6 mutation (M318V/N321A/N325S) abolished binding of mAbs 1F7 and 1F10. The mut5 mutation (A279T/A282T) reduced binding of mAb 3E3 by ∼70% (mean fluorescence data in Figure S4). Inspection of the DDR1-3E3 interface structure (Figure 5) suggests that, in order to completely disrupt 3E3 binding, Arg341 would have to be substituted in addition to Ala279 and Ala282. Given that Arg341 (mut7) interacts with Met318 (mut6), disruption of 3E3 binding is likely to require a combination of mut5-7—that is, a complete reconfiguring to the mouse structure at the base of the DS-like domain. Binding of mAb 5D5 was not affected by any of the mutations, and we assume that the 5D5 epitope similarly is a combination of the linear sequence motifs targeted in our experiments. In summary, the six inhibitory anti-DDR1 mAbs for which the epitopes could be defined (by either mutation or structure determination) bind to two distinct regions of the DS-like domain that are >50 Å away from the collagen-binding site in the DS domain (Figure 6B).

## Discussion

In this article, we report three major findings that advance the mechanistic understanding of DDR signaling: First, crystal structure analysis has revealed that the extracellular region of DDRs consists of two structurally related domains, a collagen-binding DS domain and a DS-like domain. Second, we have generated anti-DDR1 mAbs that inhibit collagen-induced DDR1 activation by binding to the DS-like domain. Third, we have identified a conserved surface patch in the DS domain that is distinct from the collagen-binding site, yet is required for DDR activation. These results are integrated into a working model of how collagen binding might alter the extracellular structure of DDRs and thereby lead to receptor activation.

The N-terminal domain of DDRs has long been recognized as a member of the DS superfamily (Johnson et al., 1993; Karn et al., 1993), and its role in collagen binding is understood in atomic detail (Carafoli et al., 2009; Ichikawa et al., 2007). Our crystal structure shows that the second DDR domain is a distant relative of the DS domain, termed the DS-like domain. Tandem repeats of DS domains occur in a number of secreted and cell surface proteins (Baumgartner et al., 1998; Kiedzierska et al., 2007). In the blood coagulation factors V and VIII, the two DS domains are arranged side by side with limited contacts between them, so that their top loops can both interact with the same cell membrane (Adams et al., 2004; Ngo et al., 2008; Shen et al., 2008). In neuropilin-1 and -2, the two DS domains are related by a ∼90° rotation and form a compact structure, as in DDR1 (Appleton et al., 2007; Vander Kooi et al., 2007). This angled arrangement in DDR1 results in the C terminus of the DS-like domain emerging near the interdomain linker. The presumably unstructured JM region of DDR1 linking the DS-like domain to the TM helix (residues 368–417) contains 12 prolines and a number of predicted *N*- and *O*-linked glycosylation sites. If fully extended, it would project the DS and DS-like domains of DDR1 ∼150 Å from the cell surface. The JM regions of other DDRs are similarly long, ranging from 32 to 74 residues.

mAbs directed against RTKs are invaluable tools for research and have been developed into successful therapeutics (Adams and Weiner, 2005). We have characterized seven anti-DDR1 mAbs that inhibit DDR1 function by binding to two distinct regions in the DS-like domain. Notably, Fab fragments derived from these mAbs were equally effective as DDR1 inhibitors. No mAbs were obtained that bind to the DS domain, possibly reflecting the higher degree of surface conservation in that domain (not shown). In agreement with their epitope locations, the mAbs inhibit DDR1 function without blocking collagen binding. We think that they do so by preventing the proximity of the two DS-like domains and the following JM regions in the collagen-bound, signaling, state of the DDR1 dimer (Noordeen et al., 2006). Deletion of the DS-like domain or JM region of DDR1 results in receptors that are not trafficked to the cell membrane, so the contribution of these regions to signaling could not be studied (Noordeen et al., 2006). Remarkably, however, the DS-like domain of DDR2 could be deleted without abrogating collagen-induced receptor autophosphorylation (Leitinger, 2003), suggesting that the DS-like domain is not making any essential contacts in the signaling DDR dimer. This leaves the collagen-bound DS domain as the most likely site of contact between the extracellular regions of the two DDR protomers in the signaling dimer.

An analysis of crystal lattice contacts in the DDR1-3E3 Fab structure led to the fortuitous discovery of functionally important residues near the base of the DS domain, close to the interface with the DS-like domain and distant from the collagen-binding site at the top of the DS domain. The patch formed by these residues is the largest concentration of conserved surface residues in the extracellular region of DDRs apart from the collagen-binding site, consistent with its essential role in signaling. We think that the conserved patch is involved in mediating protomer contacts in the signaling DDR dimer, either by forming a direct DS-DS interface or by providing a secondary collagen-binding site. The latter alternative is more appealing, because it provides a plausible mechanism whereby collagen could cross-link two DS domains (analogous to the “composite binding site” model discussed by Carafoli et al., 2009). In solution, the isolated DDR2 DS domain binds a 28-residue collagen peptide with 1:1 stoichiometry (Carafoli et al., 2009). However, inspection of the crystal lattice of this DS-collagen complex reveals that the conserved patch is involved in a lattice contact with the N-terminal glycine-proline-hydroxyproline triplets of the collagen peptide. This intriguing observation may suggest that the conserved patch in the DS domain indeed has weak affinity for collagenous sequences and, therefore, could provide a secondary collagen-binding site in dimeric, full-length DDR.

Whichever interactions are formed between the DS domains and the collagen ligand, they are expected to lead to structural changes within the DDR dimer that are propagated across the cell membrane to result in DDR autophosphorylation (Noordeen et al., 2006). Tight coupling of the extracellular conformational changes to intracellular domain arrangements is difficult to imagine in the DDRs, given their long, and presumably flexible, JM regions. Recent studies of the epidermal growth factor receptor (EGFR) have shown that the conformational coupling across the cell membrane is looser than commonly believed, even in a receptor with less extensive JM regions (Lu et al., 2010; Mi et al., 2011). However, one important difference is that the TM helices of DDRs have a much higher propensity for self-interactions than that of EGFR (Finger et al., 2009). We propose that the TM helices are largely responsible for constitutive DDR dimerization (Noordeen et al., 2006), but that collagen-induced interactions involving the DS domains are additionally required for DDR activation.

## Experimental Procedures

### DNA Constructs and Site-Directed Mutagenesis

All mutations were generated by strand overlap extension PCR using a cDNA of human DDR1 as a template (Leitinger, 2003). The PCR primers used to generate these constructs are available on request. The amplified DNAs were cloned into the mammalian expression vectors pcDNA3.1/Zeo (Invitrogen) or pRK5 (BD PharMingen) for expression of full-length proteins, or into modified pCEP vectors (Kohfeldt et al., 1997) for expression of soluble proteins. All PCR-derived DNA constructs were verified by sequencing.

### Production of Soluble DDR1 Proteins

The following proteins were produced as described elsewhere (Leitinger, 2003): His-DDR1 contains the entire extracellular region of human DDR1 (residues 19–416 of UniProt entry Q08345). His-DDR2 contains the entire extracellular region of human DDR2 (residues 22–398 of UniProt entry Q16832). His-DS-DDR1 and His-ΔDS-DDR1 are deletion constructs based on His-DDR1. His-DS-DDR1 lacks the DS-like domain (Δ201–369), and His-ΔDS-DDR1 lacks the DS domain (Δ31–185); both proteins retain the JM region. DDR1-Fc contains the entire DDR1 extracellular region fused to a C-terminal human IgG1 Fc sequence (Leitinger, 2003; Xu et al., 2011).

The DDR1 construct for crystallography contains the DS and DS-like domains of human DDR1 (residues 30–367) fused to a C-terminal His-tag (AAAHHHHHH). A vector-derived APLA sequence is present at the N terminus of the mature protein. The protein was produced in human embryonic kidney HEK293 c18 cells (ATCC). The cells were grown at 37°C with 5% CO_2_ in Dulbecco's modified Eagle's medium/F12 (Invitrogen) containing 10% fetal bovine serum, 2 mM glutamine, 10 U/ml penicillin, 100 μg/ml streptomycin, and 250 μg/ml geneticin. The cells were transfected with the pCEP-Pu expression plasmid using Fugene (Roche Diagnostics) and were selected with 1 μg/ml puromycin (Sigma). Confluent cells in a HYPERFlask (Corning) were washed twice with PBS and incubated with serum-free medium for 3–4 weeks, with weekly medium exchanges. The pooled serum-free conditioned medium was loaded onto a 5-ml HisTrap column (GE Healthcare) using an Äkta Purifier (GE Healthcare). The protein was eluted with 500 mM imidazole in PBS, concentrated using a Vivaspin centrifugal device (Sartorius), and further purified on a Superdex 200 HR10/30 size-exclusion chromatography column (GE Healthcare) with Tris-buffered saline (TBS) (25 mM Tris, 150 mM NaCl, and 2 mM KCl [pH 7.4]) as the running buffer.

### Generation of Anti-DDR1 Antibodies

To prepare an untagged antigen for immunization, 500 μg of His-DDR1 (Leitinger, 2003) was digested with 25 U EKMax enterokinase (Invitrogen) for 16 hr at 4°C. EKMax was removed with EK-Away resin (Invitrogen) according to the manufacturer's protocol. Uncleaved His-DDR1 and the cleaved tag were removed with TALON metal affinity beads (Clontech). The untagged DDR1 protein was dialyzed against PBS and concentrated to 2 mg/ml by ultrafiltration. Mouse anti-DDR1 mAbs were generated by immunizing female BALB/c mice with the untagged DDR1 protein. Three days after the final boost, one mouse was sacrificed to obtain splenocytes for hybridoma production by standard procedures. Hybridoma cell supernatants were screened against DDR1-Fc and His-DDR1 proteins by ELISA. Reactive hybridoma supernatants were further screened for recognition of native DDR1 by cell-based ELISA, using HEK293 cells expressing full-length DDR1. Positive hybridoma cells were subcloned by limited dilution and screened as above. The isotype of each mAb was determined by standard methods. All mAbs are of the IgG1 isotype, with the exception of mAb 1F10, which is IgG2b.

### Antibody and Fab Fragment Production

Hybridoma cells were grown at 37°C with 5% CO_2_ in RPMI-1640 medium (Invitrogen) containing 10% fetal bovine serum, 1 mM sodium pyruvate, 10 U/ml penicillin, 100 μg/ml streptomycin, and 1 μg/ml fungizone (Invitrogen). The serum concentration was gradually reduced to 5% in a final culture volume of 1 l. The hybridoma cell culture supernatant was loaded onto a 2 × 1-ml HiTrap rProtein A column (GE Healthcare). The mAbs were eluted with Immunopure gentle elution buffer (Pierce) and dialyzed against TBS. Fab fragments were generated with a Fab Preparation Kit (Pierce) according to the manufacturer's protocol. Briefly, 8 mg of mAb were incubated overnight at 37°C with activated papain immobilized on agarose resin. The Fc fragment and undigested mAb were removed using a 1-ml HiTrap rProtein A column (GE Healthcare), yielding ∼3 mg of Fab fragment. The Fab fragments used in cocrystallization experiments were further purified by size-exclusion chromatography on a Superdex 200 HR10/30 column (GE Healthcare) with TBS as the running buffer.

### mAb cDNA Synthesis and Sequencing

Total RNA was prepared from ∼10^7^ 3E3 hybridoma cells using the RNeasy Mini Kit (QIAGEN). The RNA was reverse-transcribed, and cDNA fragments encoding the heavy- and light-chain variable regions of the mAb were amplified using the SuperScript III One-Step RT-PCR system (Invitrogen) and suitable universal primers (Orlandi et al., 1989). The PCR products were gel-purified and sequenced using the same primers. The mAb residues are numbered according to Al-Lazikani et al. (1997).

### DDR1 Activation Assay

The assay was performed as described elsewhere (Leitinger, 2003). Briefly, HEK293 cells were grown in 12-well tissue culture plates and transfected with 2 μg/well of DDR1 wild-type or mutant plasmid DNA using calcium phosphate precipitation. Twenty-four hours after transfection, the cells were incubated with serum-free medium for 16 hr. Cells were then stimulated with 10–50 μg/ml acid-soluble rat tail collagen I (Sigma) for 90 min at 37°C before being lysed. In the inhibition experiments, anti-DDR1 mAbs or their Fab fragments were added together with collagen I, without prior incubation. Aliquots of the cell lysates were subjected to SDS-PAGE and blotted onto nitrocellulose membranes. The blots were first probed with a mouse anti-phosphotyrosine mAb (clone 4G10, Upstate Biotechnology) followed by a horseradish peroxidase-conjugated sheep anti-mouse Ig (Amersham Biosciences). Detection was done by Enhanced Chemiluminescence Plus (Amersham Biosciences) using an Ettan DIGE Imager (GE Healthcare). To reprobe the blots, the membranes were treated with Ab stripping solution (Alpha Diagnostic International), followed by incubation with rabbit anti-DDR1 Ab (SC-532, Santa Cruz Biotechnology), and finally goat horseradish peroxidase-conjugated anti-rabbit Ig (P0448, DAKO).

### ELISA and Solid Phase Binding Assays

Recombinant DDR proteins, diluted to 10 μg/ml in 50 mM Tris and 100 mM NaCl (pH 8.5), were coated in 50-μl aliquots onto Maxisorp 96-well plates (Nalgene NUNC) overnight at room temperature. The wells were blocked with 150 μl of incubation buffer (PBS containing 40 μg/ml bovine milk κ-casein [Sigma] and 0.05% Tween-20) for 1 hr at room temperature. Anti-DDR1 mAbs were added at 30 μg/ml in 50-μl aliquots and were incubated for 1.5 hr at room temperature. The wells were washed six times with incubation buffer, followed by the addition of horseradish peroxidase-conjugated sheep anti-mouse Ig (Amersham Biosciences, 1:1000 dilution in incubation buffer) for 1.5 hr at room temperature. After six washes as above, bound mAbs were detected with 75 μl/well of 500 μg/ml o-phenylenediamine dihydrochloride (Sigma-Aldrich) in 50 mM citrate-phosphate (pH 5.0). The reaction was stopped after 3–5 min with 50 μl/well of 3 M H_2_SO_4_. The absorbance at 492 nm was measured using a Sunrise 96-well plate reader (Tecan).

To measure DDR1 binding to the collagen-derived peptide III-23, Immulon 2 HB 96-well plates (Fisher Scientific) were coated overnight at room temperature with 10 μg/ml III-23 in 10 mM acetic acid (Xu et al., 2011). The wells were then blocked in incubation buffer as described above. DDR1-Fc proteins were added in various concentrations and incubated for 3 hr. In the inhibition experiments, the DDR1-Fc protein was incubated with anti-DDR1 mAbs for 30 min at room temperature before being added to the wells. After six washes with incubation buffer, bound DDR1-Fc was detected with horseradish peroxidase-conjugated goat anti-human Fc (Jackson ImmunoResearch Laboratories, 1:3333 dilution), added for 1 hr at room temperature. The assay was completed as described above.

### Flow Cytometry

HEK293 cells were grown in 6-well plates and transfected with 5 μg/well of DDR1 wild-type or mutant plasmid DNA using calcium phosphate precipitation; 48 hr after transfection, the cells were dissociated with nonenzymatic cell dissociation solution (Sigma) and resuspended in PBS containing 1% BSA. The cells were incubated with primary mAb or mouse IgG1 isotype control Ab (Cambridge Bioscience) at 10 μg/ml in 100 μl PBS/BSA for 30 min on ice, followed by three washes with PBS/BSA and incubation with FITC-conjugated goat anti-mouse IgG (F-9006, Sigma) for 30 min on ice. After three washes as above, the cells were resuspended in 2% formaldehyde in PBS and analyzed on a FACS Calibur flow cytometer using Cell Quest Pro software (Becton Dickinson Biosciences).

### Crystal Structure Determination

The purified DDR1 protein for crystallography and the 3E3 Fab fragment were mixed in an equimolar ratio and incubated on ice for 30 min. The solution was subjected to size-exclusion chromatography on a Superdex 200 HR10/30 column (GE Healthcare) with TBS as the running buffer. The DDR1-3E3 Fab complex was eluted as a single peak and was concentrated to 6 mg/ml. Sitting drop vapor diffusion crystallization screens were set up using a Mosquito nanolitre robot (TTP LabTech). Crystals were obtained after 1–2 days at room temperature using 2% Tacsimate (pH 5.0) (Hampton Research), 100 mM sodium citrate tribasic dihydrate (pH 5.6), and 20% PEG 3350 as precipitant. Crystals were flash-frozen in liquid nitrogen after a brief soak in mother liquor supplemented with 25% glycerol. Diffraction data were collected at 100 K on station I02 at the Diamond Light Source (Oxfordshire, UK). The data were processed with MOSFLM (www.mrc-lmb.cam.ac.uk/harry/mosflm) and programs of the CCP4 suite (CCP4, 1994). The DDR1-3E3 Fab structure was solved by molecular replacement with PHASER (McCoy et al., 2007), using as search models the DDR2 DS domain (PDB entry 2wuh) and a Fab fragment of an Ab directed against neuropilin-2 (PDB entry 2qqk). The electron density map calculated from the correctly positioned search models showed weak density for several β strands in the DS-like domain, which were used to place the related coagulation factor V/VIII type C domain of galactose oxidase (PDB entry 1k3i) as an aid for model building. The model was built with O (Jones et al., 1991) and refined with CNS (Brünger et al., 1998). Crystallographic statistics are summarized in Table 1. The figures were made with PyMOL (http://www.pymol.org).

## Figures and Tables

**Table 1:** Crystallographic Statistics of the DDR1-3E3 Fab Structure

Data Collection	Value
Space group	C222_1_
Unit cell dimensions	
a, b, c (Å)	102.51, 251.48, 75.37
α, β, γ (°)	90, 90, 90
Asymmetric unit content	1:1 DDR1-3E3 Fab complex
Solvent content (%)	57
Resolution (Å)	50–2.8 (2.95–2.80)^a^
R_merge_	0.079 (0.429)
<I/σ(I) >	14.3 (3.8)
Completeness (%)	98.7 (98.9)
Multiplicity	6.0 (6.1)

**Refinement**	

Resolution (Å)	20–2.8
Reflections	24035
Protein atoms	2700 (DDR1) + 3169 (3E3 Fab)
Solvent atoms	2 Ca^2+^ + 14 H_2_O
R_work_/R_free_	0.216/0.286
Rmsd bonds (Å)	0.007
Rmsd angles (°)	1.4
Average B-factor (Å^2^)	53.6
Ramachandran plot (%)^b^	91.3/98.1

a Values in parentheses are for the highest resolution shell.

b Percentage of residues in favored and allowed regions (Chen et al., 2010).

## Accession Number

The coordinates of the DDR1-3E3 Fab structure have been deposited in the Protein Data Bank (entry code 4ag4).

## References

[bib1] Abdulhussein R., Koo D.H., Vogel W.F. (2008). Identification of disulfide-linked dimers of the receptor tyrosine kinase DDR1. J. Biol. Chem..

[bib2] Adams G.P., Weiner L.M. (2005). Monoclonal antibody therapy of cancer. Nat. Biotechnol..

[bib3] Adams T.E., Hockin M.F., Mann K.G., Everse S.J. (2004). The crystal structure of activated protein C-inactivated bovine factor Va: implications for cofactor function. Proc. Natl. Acad. Sci. USA.

[bib4] Ali B.R., Xu H., Akawi N.A., John A., Karuvantevida N.S., Langer R., Al-Gazali L., Leitinger B. (2010). Trafficking defects and loss of ligand binding are the underlying causes of all reported DDR2 missense mutations found in SMED-SL patients. Hum. Mol. Genet..

[bib5] Al-Lazikani B., Lesk A.M., Chothia C. (1997). Standard conformations for the canonical structures of immunoglobulins. J. Mol. Biol..

[bib6] Appleton B.A., Wu P., Maloney J., Yin J., Liang W.C., Stawicki S., Mortara K., Bowman K.K., Elliott J.M., Desmarais W. (2007). Structural studies of neuropilin/antibody complexes provide insights into semaphorin and VEGF binding. EMBO J..

[bib7] Bargal R., Cormier-Daire V., Ben-Neriah Z., Le Merrer M., Sosna J., Melki J., Zangen D.H., Smithson S.F., Borochowitz Z., Belostotsky R., Raas-Rothschild A. (2009). Mutations in DDR2 gene cause SMED with short limbs and abnormal calcifications. Am. J. Hum. Genet..

[bib8] Baumgartner S., Hofmann K., Chiquet-Ehrismann R., Bucher P. (1998). The discoidin domain family revisited: new members from prokaryotes and a homology-based fold prediction. Protein Sci..

[bib9] Boraston A.B., Bolam D.N., Gilbert H.J., Davies G.J. (2004). Carbohydrate-binding modules: fine-tuning polysaccharide recognition. Biochem. J..

[bib10] Brünger A.T., Adams P.D., Clore G.M., DeLano W.L., Gros P., Grosse-Kunstleve R.W., Jiang J.S., Kuszewski J., Nilges M., Pannu N.S. (1998). Crystallography & NMR system: a new software suite for macromolecular structure determination. Acta Crystallogr. D Biol. Crystallogr..

[bib11] Carafoli F., Bihan D., Stathopoulos S., Konitsiotis A.D., Kvansakul M., Farndale R.W., Leitinger B., Hohenester E. (2009). Crystallographic insight into collagen recognition by discoidin domain receptor 2. Structure.

[bib12] Chen V.B., Arendall W.B., Headd J.J., Keedy D.A., Immormino R.M., Kapral G.J., Murray L.W., Richardson J.S., Richardson D.C. (2010). MolProbity: all-atom structure validation for macromolecular crystallography. Acta Crystallogr. D Biol. Crystallogr..

[bib13] Collaborative Computational Project, Number 4 (1994). The CCP4 suite: programs for protein crystallography. Acta Crystallogr. D Biol. Crystallogr..

[bib14] Finger C., Escher C., Schneider D. (2009). The single transmembrane domains of human receptor tyrosine kinases encode self-interactions. Sci. Signal..

[bib15] Gschwind A., Fischer O.M., Ullrich A. (2004). The discovery of receptor tyrosine kinases: targets for cancer therapy. Nat. Rev. Cancer.

[bib16] Ichikawa O., Osawa M., Nishida N., Goshima N., Nomura N., Shimada I. (2007). Structural basis of the collagen-binding mode of discoidin domain receptor 2. EMBO J..

[bib17] Ikeda K., Wang L.H., Torres R., Zhao H., Olaso E., Eng F.J., Labrador P., Klein R., Lovett D., Yancopoulos G.D. (2002). Discoidin domain receptor 2 interacts with Src and Shc following its activation by type I collagen. J. Biol. Chem..

[bib18] Johnson J.D., Edman J.C., Rutter W.J. (1993). A receptor tyrosine kinase found in breast carcinoma cells has an extracellular discoidin I-like domain. Proc. Natl. Acad. Sci. USA.

[bib19] Jones T.A., Zou J.Y., Cowan S.W., Kjeldgaard M. (1991). Improved methods for building protein models in electron density maps and the location of errors in these models. Acta Crystallogr. A.

[bib20] Karn T., Holtrich U., Bräuninger A., Böhme B., Wolf G., Rübsamen-Waigmann H., Strebhardt K. (1993). Structure, expression and chromosomal mapping of TKT from man and mouse: a new subclass of receptor tyrosine kinases with a factor VIII-like domain. Oncogene.

[bib21] Kiedzierska A., Smietana K., Czepczynska H., Otlewski J. (2007). Structural similarities and functional diversity of eukaryotic discoidin-like domains. Biochim. Biophys. Acta.

[bib22] Kohfeldt E., Maurer P., Vannahme C., Timpl R. (1997). Properties of the extracellular calcium binding module of the proteoglycan testican. FEBS Lett..

[bib23] Konitsiotis A.D., Raynal N., Bihan D., Hohenester E., Farndale R.W., Leitinger B. (2008). Characterization of high affinity binding motifs for the discoidin domain receptor DDR2 in collagen. J. Biol. Chem..

[bib24] Krissinel E., Henrick K. (2004). Secondary-structure matching (SSM), a new tool for fast protein structure alignment in three dimensions. Acta Crystallogr. D Biol. Crystallogr..

[bib25] Labrador J.P., Azcoitia V., Tuckermann J., Lin C., Olaso E., Mañes S., Brückner K., Goergen J.L., Lemke G., Yancopoulos G. (2001). The collagen receptor DDR2 regulates proliferation and its elimination leads to dwarfism. EMBO Rep..

[bib26] Lawrence M.C., Colman P.M. (1993). Shape complementarity at protein/protein interfaces. J. Mol. Biol..

[bib27] Leitinger B. (2003). Molecular analysis of collagen binding by the human discoidin domain receptors, DDR1 and DDR2: identification of collagen binding sites in DDR2. J. Biol. Chem..

[bib28] Leitinger B. (2011). Transmembrane collagen receptors. Annu. Rev. Cell Dev. Biol..

[bib29] Lemmon M.A., Schlessinger J. (2010). Cell signaling by receptor tyrosine kinases. Cell.

[bib30] Lu C., Mi L.Z., Grey M.J., Zhu J., Graef E., Yokoyama S., Springer T.A. (2010). Structural evidence for loose linkage between ligand binding and kinase activation in the epidermal growth factor receptor. Mol. Cell. Biol..

[bib31] McCoy A.J., Grosse-Kunstleve R.W., Adams P.D., Winn M.D., Storoni L.C., Read R.J. (2007). Phaser crystallographic software. J. Appl. Cryst..

[bib32] Mi L.Z., Lu C., Li Z., Nishida N., Walz T., Springer T.A. (2011). Simultaneous visualization of the extracellular and cytoplasmic domains of the epidermal growth factor receptor. Nat. Struct. Mol. Biol..

[bib33] Mihai C., Chotani M., Elton T.S., Agarwal G. (2009). Mapping of DDR1 distribution and oligomerization on the cell surface by FRET microscopy. J. Mol. Biol..

[bib34] Nettleship J.E., Ren J., Rahman N., Berrow N.S., Hatherley D., Barclay A.N., Owens R.J. (2008). A pipeline for the production of antibody fragments for structural studies using transient expression in HEK 293T cells. Protein Expr. Purif..

[bib35] Ngo J.C., Huang M., Roth D.A., Furie B.C., Furie B. (2008). Crystal structure of human factor VIII: implications for the formation of the factor IXa-factor VIIIa complex. Structure.

[bib36] Noordeen N.A., Carafoli F., Hohenester E., Horton M.A., Leitinger B. (2006). A transmembrane leucine zipper is required for activation of the dimeric receptor tyrosine kinase DDR1. J. Biol. Chem..

[bib37] Orlandi R., Güssow D.H., Jones P.T., Winter G. (1989). Cloning immunoglobulin variable domains for expression by the polymerase chain reaction. Proc. Natl. Acad. Sci. USA.

[bib38] Shen B.W., Spiegel P.C., Chang C.H., Huh J.W., Lee J.S., Kim J., Kim Y.H., Stoddard B.L. (2008). The tertiary structure and domain organization of coagulation factor VIII. Blood.

[bib39] Shrivastava A., Radziejewski C., Campbell E., Kovac L., McGlynn M., Ryan T.E., Davis S., Goldfarb M.P., Glass D.J., Lemke G., Yancopoulos G.D. (1997). An orphan receptor tyrosine kinase family whose members serve as nonintegrin collagen receptors. Mol. Cell.

[bib40] Vander Kooi C.W., Jusino M.A., Perman B., Neau D.B., Bellamy H.D., Leahy D.J. (2007). Structural basis for ligand and heparin binding to neuropilin B domains. Proc. Natl. Acad. Sci. USA.

[bib41] Vogel W., Gish G.D., Alves F., Pawson T. (1997). The discoidin domain receptor tyrosine kinases are activated by collagen. Mol. Cell.

[bib42] Vogel W.F., Aszódi A., Alves F., Pawson T. (2001). Discoidin domain receptor 1 tyrosine kinase has an essential role in mammary gland development. Mol. Cell. Biol..

[bib43] Vogel W.F., Abdulhussein R., Ford C.E. (2006). Sensing extracellular matrix: an update on discoidin domain receptor function. Cell. Signal..

[bib44] Xu H., Raynal N., Stathopoulos S., Myllyharju J., Farndale R.W., Leitinger B. (2011). Collagen binding specificity of the discoidin domain receptors: binding sites on collagens II and III and molecular determinants for collagen IV recognition by DDR1. Matrix Biol..

