# Supplementary material for: Structure of the Discoidin Domain Receptor 1 Extracellular Region Bound to an Inhibitory Fab Fragment Reveals Features Important for Signaling
Source: Structure. 2012 Apr 4;20-178(4):688–97. doi: 10.1016/j.str.2012.02.011 (PMC3320712; doi:10.1016/j.str.2012.02.011)
Supplement: Document S1. Figures S1–S4 [file mmc1.pdf]

Structure, Volume *20*

Supplemental Information

Structure of the Discoidin Domain Receptor 1 Extracellular Region Bound to an Inhibitory Fab Fragment Reveals Features Important for Signaling

Federico Carafoli, Marie Cathrin Mayer, Kazushige Shiraishi, Mira Anguelova Pecheva, Lai Yi Chan, Ruodan Nan, Birgit Leitinger, and Erhard Hohenester

Inventory of Supplemental Information

Figure S1: related to Figure 1.

Figure S2: related to Figure 3.

Figure S3: related to Figure 3.

Figure S4: related to Figure 6.

Figure S1

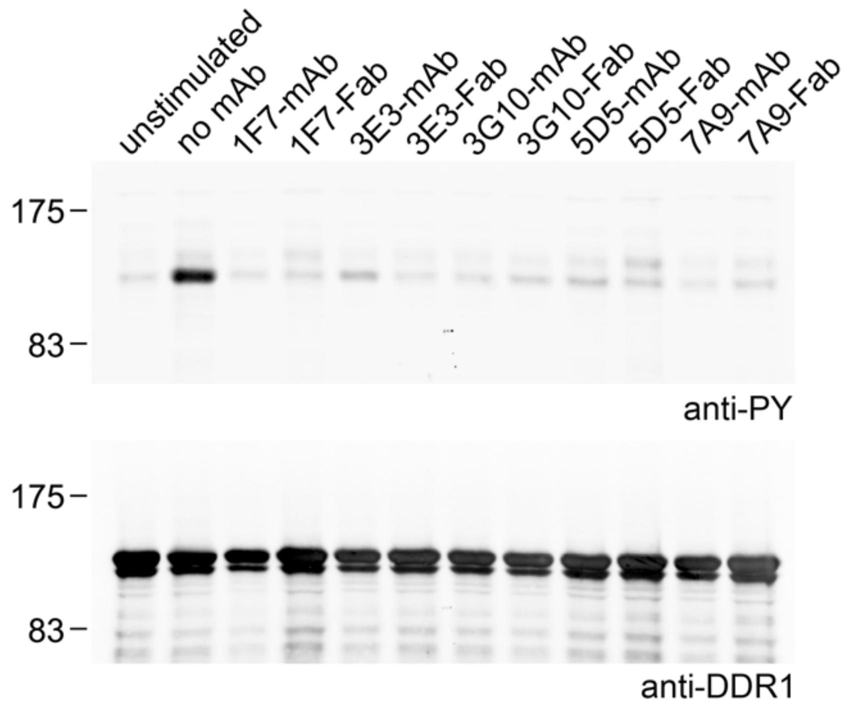
unstimulated
no mAb
1F7-mAb
1F7-Fab
3E3-mAb
3E3-Fab
3G10-mAb
3G10-Fab
5D5-mAb
5D5-Fab
7A9-mAb
7A9-Fab
175
83
anti-PY
175
83
anti-DDR1

Figure S2

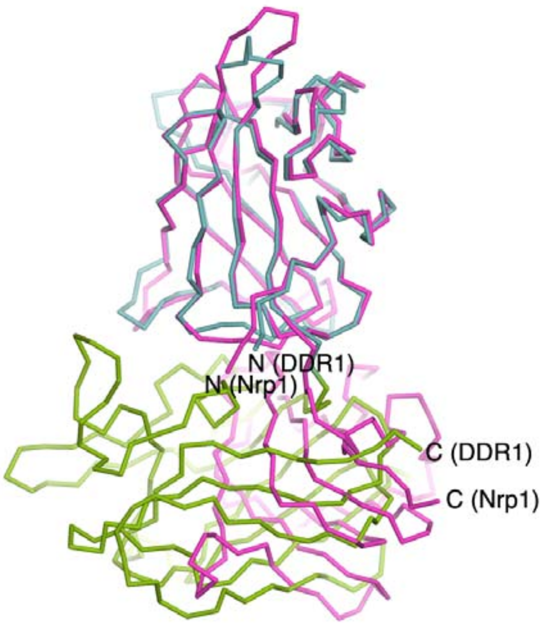

Figure S3

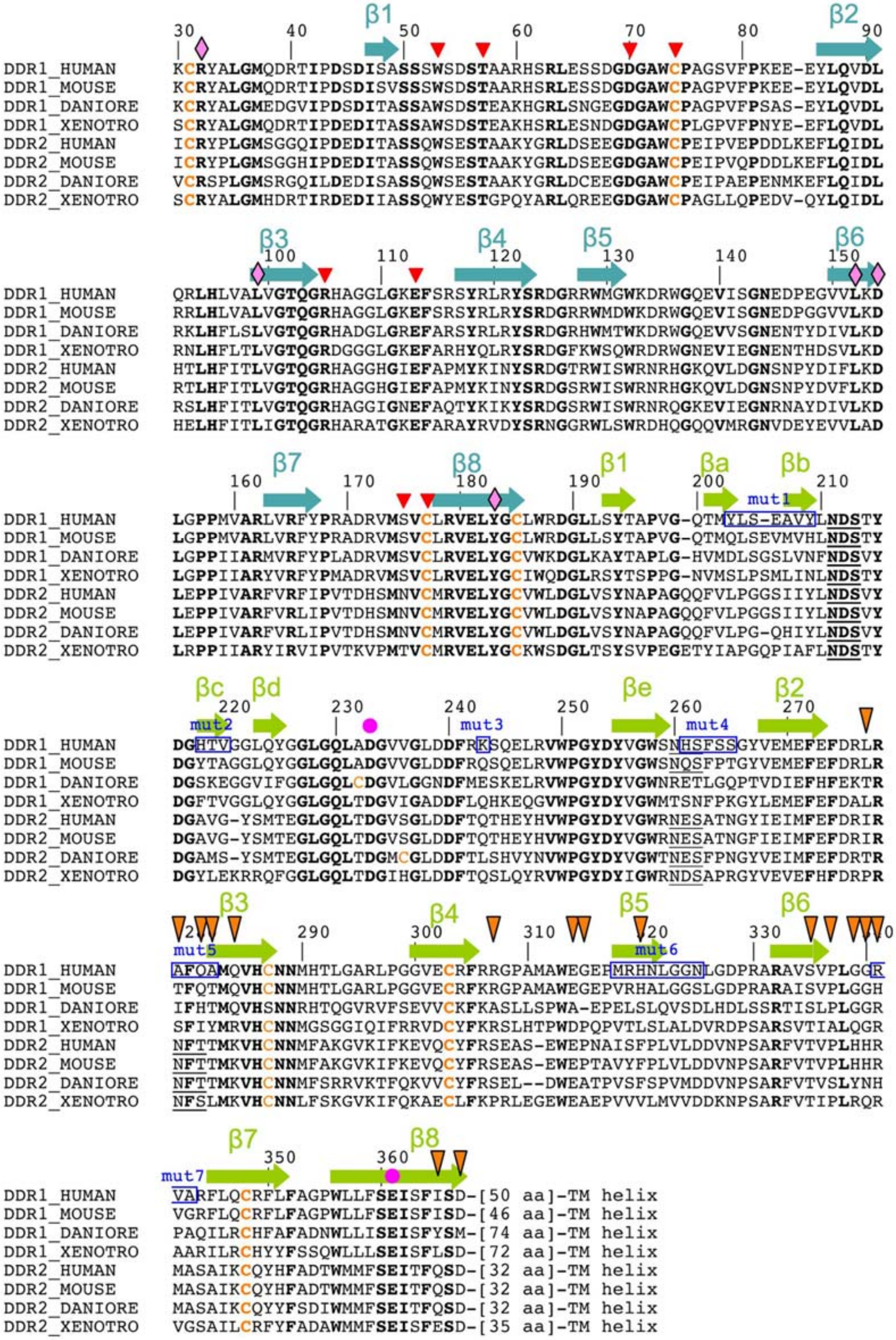
β1 β2 β3 β4 β5 β6 β7 β8
βa βb βc βd βe
mut1 mut2 mut3 mut4 mut5 mut6 mut7
30 40 50 60 70 80 90
DDR1_HUMAN KCRYALGMQDRTIPDSDISASSSWSDSTAARHSRLESSDGDGAWCPAGSVFPKEE-EYLQVDL
DDR1_MOUSE KCRYALGMQDRTIPDSDISVSSSWSDSTAARHSRLESSDGDGAWCPAGPVFPKEE-EYLQVDL
DDR1_DANIORE KCRYALGMEDGVIPDSDITASSAWSDSTEAKHGRLSNGEGDGAWCPAGPVFPSAS-EYLQVDL
DDR1_XENOTRO SCRYALGMQDRTIPDEDITASSAWSDSTEAKHSRLESNDGDGAWCPLGPVFPNYE-EFLQVDL
DDR2_HUMAN ICRYPLGMSGGQIPDEDITASSQWSESTAAKYGRLDSEEGDGAWCPEIPVEPDDLKEFLQIDL
DDR2_MOUSE ICRYPLGMSGGHIPDEDITASSQWSESTAAKYGRLDSEEGDGAWCPEIPVQPDDLKEFLQIDL
DDR2_DANIORE VCRSPLGMSRGQILDEDISASSQWSESTAAKYGRLDCEEGDGAWCPEIPAEPENMKEFLQIDL
DDR2_XENOTRO SCRYALGMHDRTIRDEDIIASSQWYESTGPQYARLQREEGDGAWCPAGLLQPEDV-QYLQIDL
100 110 120 130 140 150
DDR1_HUMAN QRLHLVALVGTQGRHAGGLGKEFSRSYRLRYSRDGRRWMGWKDRWGQEVISGNEDPEGVVLKD
DDR1_MOUSE RRLHLVALVGTQGRHAGGLGKEFSRSYRLRYSRDGRRWMDWKDRWGQEVISGNEDPGGVVLKD
DDR1_DANIORE RKLHFLSLVGTQGRHADGLGREFARSYRLRYSRDGRHWMTWKDRWGQEVVSGNENTYDIVLKD
DDR1_XENOTRO RNLHFLTLVGTQGRDGGGLGKEFARHYQLRYSRDGFKWSQWRDRWGNEVIEGNENTHDSVLKD
DDR2_HUMAN HTLHFITLVGTQGRHAGGHGIEFAPMYKINYSRDGTRWISWRNRHGKQVLDGNSNPYDIFLKD
DDR2_MOUSE RTLHFITLVGTQGRHAGGHGIEFAPMYKINYSRDGSRWISWRNRHGKQVLDGNSNPYDVFLKD
DDR2_DANIORE RSLHFITLVGTQGRHAGGIGNEFAQTYKIKYSRDGSRWISWRNRQGKEVIEGNRNAYDIVLKD
DDR2_XENOTRO HELHFITLIGTQGRHARATGKEFARAYRVDYSRNGGRWLSWRDHQGQQVMRGNVDEYEVVLAD
160 170 180 190 200 210
DDR1_HUMAN LGPPMVARLVRFYPRADRVMSVCLRVELYGCLWRDGLLSYTAPVG-QTMYLS-EAVYLNDSTY
DDR1_MOUSE LGPPMVARLVRFYPRADRVMSVCLRVELYGCLWRDGLLSYTAPVG-QTMQLSEVMVHLNDSTY
DDR1_DANIORE LGPPIIARMVRFYPLADRVMSVCLRVELYGCVWKDGLKAYTAPLG-HVMDLSGSLVNFNDSVY
DDR1_XENOTRO LGPPIIARYVRFYPMADRVMSVCLRVELYGCIWQDGLRSYTSPPG-NVMSLPSMLINLNDSTY
DDR2_HUMAN LEPPIVARFVRFIPVTDHSMNVCMRVELYGCVWLDGLVSYNAPAGQQFVLPGGSIIYLNDSVY
DDR2_MOUSE LEPPIVARFVRLIPVTDHSMNVCMRVELYGCVWLDGLVSYNAPAGQQFVLPGGSIIYLNDSVY
DDR2_DANIORE LEPPIVARFVRLIPVTDHSMNVCMRVELYGCVWLDGLVSYNAPAGQQFVLPG-QHIYLNDSVY
DDR2_XENOTRO LRPPIIARYIRVIPVTKVPMTVCMRVELYGCKWSDGLTSYSVPEGETYIAPGQPIAFLNDSTY
220 230 240 250 260 270
DDR1_HUMAN DGHTVGGLQYGGLGQLADGVVGLDDFRKSQELRVWPGYDYVGWSNHSFSSGYVEMEFEFDRLR
DDR1_MOUSE DGYTAGGLQYGGLGQLADGVVGLDDFRQSQELRVWPGYDYVGWSNQSFPTGYVEMEFEFDRLR
DDR1_DANIORE DGSKEGGVIFGGLGQLCDGVLGGNDFMESKELRVWPGYDYVGWNRETLGQPTVDIEFHFEKTR
DDR1_XENOTRO DGFTVGGLQYGGLGQLTDGVIGADDFLQHKEQGVWPGYDYVGWMTSNFPKGYLEMEFEFDALR
DDR2_HUMAN DGAVG-YSMTEGLGQLTDGVSGLDDFTQTHEYHVWPGYDYVGWRNESATNGYIEIMFEFDRIR
DDR2_MOUSE DGAVG-YSMTEGLGQLTDGVSGLDDFTQTHEYHVWPGYDYVGWRNESATNGFIEIMFEFDRIR
DDR2_DANIORE DGAMS-YSMTEGLGQLTDGMCGLDDFTLSHVYNVWPGYDYVGWTNESFPNGYVEIMFEFDRTR
DDR2_XENOTRO DGYLEKRRQFGGLGQLTDGIHGLDDFTQSLQYRVWPGYDYIGWRNDSAPRGYVEVEFHFDRPR
290 300 310 320 330
DDR1_HUMAN AFQAMQVHCNNMHTLGARLPGGVECRFRRGPAMAWEGEPMRHNLGGNLGDPRARAVSVPLGGR
DDR1_MOUSE TFQTMQVHCNNMHTLGARLPGGVECRFKRGPAMAWEGEPVRHALGGSLGDPRARAISVPLGGH
DDR1_DANIORE IFHTMQVHSNNRHTQGVRVFSEVVCKFKASLLSPWA-EPELSLQVSDLHDLSSRTISLPLGGR
DDR1_XENOTRO SFIYMRVHCNNMGSGGIQIFRRVDCYFKRSLHTPWDPQPVTLSLALDVRDPSARSVTIALQGR
DDR2_HUMAN NFTTMKVHCNNMFAKGVKIFKEVQCYFRSEAS-EWEPNAISFPLVLDDVNPSARFVTVPLHHR
DDR2_MOUSE NFTTMKVHCNNMFAKGVKIFKEVQCYFRSEAS-EWEPTAVYFPLVLDDVNPSARFVTVPLHHR
DDR2_DANIORE NFTTMKVHCNNMFSRRVKTFQKVVCYFRSEL--DWEATPVSFSPVMDDVNPSARFVTVSLYNH
DDR2_XENOTRO NFSLMKVHCNNLFSKGVKIFQKAECLFKPRLEGEWEAEPVVVLMVVDDKNPSARFVTIPLRQR
350 360
DDR1_HUMAN VARFLQCRFLFAGPWLLFSEISFISD-[50 aa]-TM helix
DDR1_MOUSE VGRFLQCRFLFAGPWLLFSEISFISD-[46 aa]-TM helix
DDR1_DANIORE PAQILRCHFAFADNWLLISEISFYSM-[74 aa]-TM helix
DDR1_XENOTRO AARILRCHYYFSSQWLLLSEISFLSD-[72 aa]-TM helix
DDR2_HUMAN MASAIKCQYHFADTWMMFSEITFQSD-[32 aa]-TM helix
DDR2_MOUSE MASAIKCQYHFADTWMMFSEITFQSD-[32 aa]-TM helix
DDR2_DANIORE MASAIKCQYYFSDIWMMFSEITFQSD-[32 aa]-TM helix
DDR2_XENOTRO VGSAILCRFYFADAWMMFSEISFESD-[35 aa]-TM helix

Figure S4

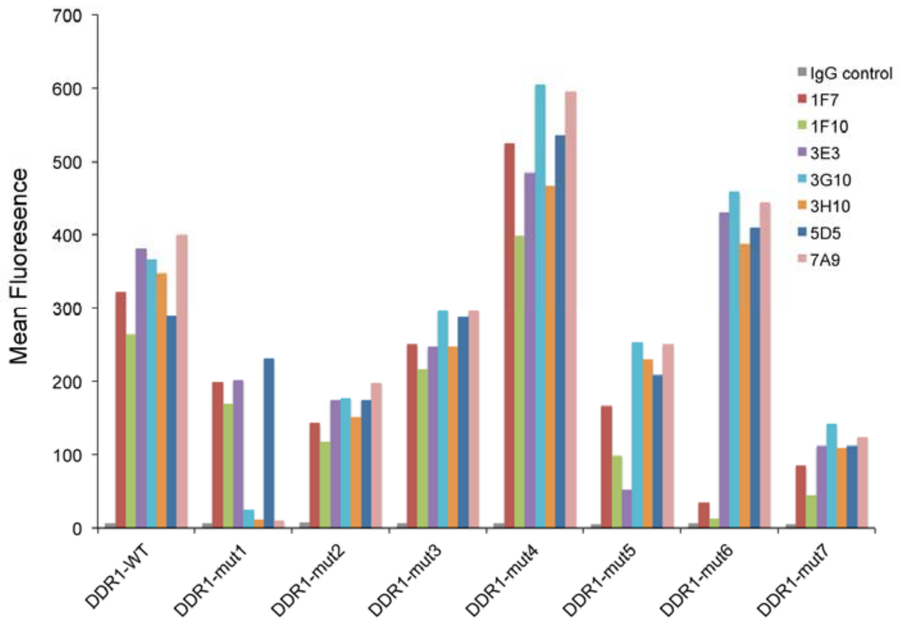
700
600
500
400
300
200
100
0
Mean Fluoresence
IgG control
1F7
1F10
3E3
3G10
3H10
5D5
7A9
DDR1-WT
DDR1-mut1
DDR1-mut2
DDR1-mut3
DDR1-mut4
DDR1-mut5
DDR1-mut6
DDR1-mut7

SUPPLEMENTAL FIGURE LEGENDS

Figure S1, related to Figure 1. Selected Fab fragments block collagen-induced DDR1 phosphorylation. DDR1b was transiently expressed in HEK293 cells. The cells were stimulated for 90 min at 37° C with 10 µg/ml collagen I in the presence or absence of the indicated anti-DDR1 mAbs or Fab fragments (at 10 µg/ml). Aliquots of cell lysates were analysed by SDS-PAGE and Western blotting. The blots were probed with anti-phosphotyrosine (anti-PY) mAb 4G10 (upper blot) and re-probed with anti-DDR1 Abs (lower blot). The experiment was performed three times with very similar results.

Figure S2, related to Figure 3. Superposition of DDR1 (cyan, DS domain; green DS-like domain) and the DS domain pair of neuropilin-1 (Nrp1, magenta) (Vander Kooi et al., 2007). The DDR1 DS domain was fitted to the first DS domain (b1) of Nrp1. The DDR1 DS-like domain and the second DS domain (b2) of Nrp1 are related by a ~60° rotation about a vertical axis.

Figure S3, related to Figure 3. Alignment of selected DDR1 and DDR2 sequences (*Homo sapiens* DDR1, Q08345; *Mus musculus* DDR1, Q03146; *Danio rerio* DDR1, XP_001345829; *Xenopus tropicalis* DDR1, XP_002939505; *Homo sapiens* DDR2, Q16832; *Mus musculus* DDR2, Q62371; *Danio rerio* DDR2, XP_684261; *Xenopus tropicalis* DDR2, XP_002933824). The numbers above the alignment refer to the human DDR1 sequence. Conserved residues are in bold and cysteines are in orange. Predicted *N*-linked glycosylation sites are underlined. The secondary structure elements of the DDR1 structure are indicated above the alignment. Red inverted triangles indicate key collagen-binding residues in DDR2 (Carafoli et al., 2009). Magenta filled circles indicate calcium ligands in DDR1. Pink

diamonds indicate residues contributing to the conserved surface patch in the DS domain. Orange inverted triangles indicate residues involved in 3E3 Fab binding (>10 $Å^2$ reduction in solvent accessibility upon Fab binding). The seven linear and non-conservative human-to-mouse substitutions in the DS-like domain are boxed in blue and labelled mut1-7.

Figure S4, related to Figure 6. Mean fluorescence values of the flow cytometry data shown in **Figure 6A**. DDR1b wild-type or the indicated DDR1 mutants were transiently expressed in HEK293 cells. The cells were stained on ice with 10 μg/ml of the indicated anti-DDR1 mAbs or mouse IgG1 isotype control Ab followed by FITC-conjugated goat-anti mouse IgG and analysis by flow cytometry.
